# Placenta-derived extracellular vesicles in fetal health: emerging insights into brain development and environmental interactions

**DOI:** 10.1038/s12276-025-01601-2

**Published:** 2025-12-17

**Authors:** Ethan Lewis, So Jeong Lee, Hae-Ryung Park

**Affiliations:** 1 Department of Environmental Medicine, School of Medicine and Dentistry, University of Rochester, Rochester, NY USA; 2 Department of Physiology, School of Medicine, Chung-Ang University, Seoul, Republic of Korea

**Keywords:** Developmental biology, Biomarkers, Diseases, Cell biology

## Abstract

Placenta-derived extracellular vesicles (EVs) are emerging as critical regulators of maternal–fetal communication during pregnancy. These lipid bilayer-enclosed particles, primarily secreted by trophoblasts, transport bioactive cargos—including RNAs, proteins, lipids and neurotransmitters—that influence a wide range of developmental and immunological processes. While much attention has been given to their roles in maternal adaptation and health outcomes, recent studies highlight their direct impact on fetal development, particularly fetal brain development. Emerging evidence suggests that placental EVs may traverse both the placental and blood–brain barriers, thereby contributing to signaling processes that influence neurogenesis, cell fate specification and regional brain patterning. Their cargo composition is dynamic, modulated by gestational age and environmental factors such as air pollution, viral infection and chemical toxicants. These stressors can alter EV secretion and molecular content, contributing to adverse fetal outcomes including impaired organogenesis and neurodevelopmental delays. In this review, we synthesize current knowledge on placental EV biology, examine their roles in maternal and fetal health with an emphasis on neurodevelopment and evaluate how environmental exposures reshape EV-mediated signaling. We also discuss emerging technologies and translational opportunities, including EV-based diagnostics and therapeutic delivery systems. Collectively, placenta-derived EVs represent a vital yet underexplored mechanism in fetal programming, offering novel insights into the developmental origins of health and disease.

## Placental extracellular vesicles influence fetal brain development

Extracellular vesicles (EVs) are tiny particles released by cells that help them communicate. They carry important materials such as proteins and RNA, reflecting the health of the cells they come from. This review focuses on EVs from the placenta, which play a crucial role during pregnancy. These placental EVs help in communication between the mother and fetus, affecting immune responses and fetal development. The study summarized various methods to isolate and analyze these EVs, including advanced techniques such as high-resolution flow cytometry. Researchers stressed that placental EVs can cross barriers between mother and fetus, carrying signals that influence fetal organ development, including the brain. They also emphasized that environmental factors such as pollution can change the content of these EVs, potentially affecting fetal health. The findings suggest that placental EVs are vital for healthy pregnancy outcomes.

This summary was initially drafted using artificial intelligence, then revised and fact-checked by the author.

## Introduction

Extracellular vesicles (EVs) are lipid bilayer-enclosed particles secreted by nearly all cell types. They carry diverse cargos—including nucleic acids, proteins and lipids—and serve as important mediators of intercellular communication^1–4^. Their composition reflects the physiological or pathological state of the originating cells, making them central to a wide range of biological processes^5–7^. EVs participate in vascular regulation (for example, blood coagulation)^8–10^, angiogenesis^11^, immune modulation^12–14^ and reticulocyte maturation^2^. They also play essential roles in pathological conditions such as inflammation^12^ and cancer^15^.

EVs are typically categorized into three major types based on their biogenesis: exosomes (originating from multivesicular bodies), microvesicles (formed via outward budding from the plasma membrane) and apoptotic bodies (released from dying cells)^15^ (Table 1). In addition to these well-established categories, recent studies have described novel EV subtypes that further expand the complexity of the EV landscape. These include ARRDC1-mediated microvesicles (ARMMs), which bud directly from the plasma membrane through the coordinated action of arrestin domain-containing protein 1 (ARRDC1) and tumor susceptibility gene 101 (TSG101)^16,17^. Other newly recognized subtypes include migrasomes, which are released from retraction fibers of migrating cells^18,19^, exophers, which facilitate the extrusion of damaged mitochondria and aggregated proteins^20,21^, and autophagic vesicles formed during autophagy^22^. These emerging vesicle types further underscore the heterogeneity and functional diversity within the EV population (Table 1). However, a clear consensus on EV classification remains elusive due to the inherent heterogeneity in their size, content and cellular origin. In response to this challenge, international initiatives—most notably by the International Society for Extracellular Vesicles (ISEV)—are actively developing more comprehensive classification systems that integrate parameters such as vesicle size, molecular cargo and surface markers^23^. Given the existence of several in-depth reviews on EV classification and characterization^24–26^, these topics will not be covered in detail in this Review.

**Table 1:** Classification of EVs.

Type	Origin	Size (nm)	Marker proteins	References
Exosome	Endosomal multivesicular Bodies	30–150	CD9, CD63, CD81	^86,87^
Microvesicle	Plasma membrane	100–1000	Annexin V, integrins	^87,88^
Apoptotic body	Apoptotic cell disassembly	>1000	Histones, DNA fragments	^87,89^
ARMMs	Plasma membrane	40–100	ARRDC1, TSG101	^16,17,79,80^
Migrasome	Migrating cells retraction fibers	500–3000	TSPAN4	^18,19^
Exopher	Jettisoned from cell body	1000–7800	LC3	^20,21^
Autophagic EV	Autophagy	40–1000	LC3B-PE, p62	^22,87^

Among the diverse populations of EVs, placenta-derived EVs are uniquely substantial during pregnancy. Secreted by trophoblasts—particularly the syncytiotrophoblasts—placental EVs facilitate maternal–fetal communication by traversing biological barriers and influencing immune tolerance^27^, vascular development^28,29^ and maternal metabolic state^30,31^. While considerable research has focused on their impact on maternal physiology^32,33^, the potential of placental EVs to influence fetal development, especially fetal brain development, has only recently emerged as a major area of interest. This Review will (1) summarize current knowledge of placental EV biology and their roles in maternal health and pregnancy outcomes, (2) explore their roles in fetal health, with particular emphasis on neurodevelopment, (3) examine how environmental exposures shape placental EV composition and function, and (4) highlight key knowledge gaps and propose future directions for research.

## Placental EVs and maternal–fetal health outcomes

### Placental EVs—overview

Placental EVs are a unique and functionally diverse subset of EVs secreted by placental cells, most notably trophoblasts, which constitute the key interface between maternal and fetal tissues^34–36^. Advances in high-resolution flow cytometry have revealed that they predominantly range from 50 nm to 150 nm in diameter^37^. Among trophoblast subtypes, syncytiotrophoblasts—the multinucleated layer that directly contacts maternal blood—are the predominant source of EVs, releasing them continuously into maternal circulation throughout pregnancy^38^. According to RNA sequencing data, syncytiotrophoblasts are the second most abundant placental cell type, following cytotrophoblasts and preceding mesenchymal stromal cells^39^. However, it is important to note that current single-cell sequencing technologies may underrepresent the true number of syncytiotrophoblasts owing to technical limitations associated with capturing large, multinucleated cells.

The biological relevance of placental EVs has been demonstrated in multiple models. A landmark study by Sheller-Miller et al. used a cyclic recombinase fluorescent reporter mouse model, wherein fetal tissues expressed fluorescently labeled EV cargo^40^. These vesicles were subsequently detected in maternal blood, confirming fetal-to-maternal EV transfer. In a complementary experiment, maternal injection of engineered EVs with a tissue-specific fluorescent reporter led to altered fluorescence in fetal tissues, demonstrating maternal-to-fetal transfer of vesicles across the placental barrier and direct interaction and modulation of fetal physiology. This bidirectional trafficking underscores the role of placental EVs as active mediators of maternal–fetal communication^38,41^.

#### Surface markers of placental EVs

Characterizing placental EVs remains technically challenging due to their heterogeneous size, cellular origin and molecular composition. While general exosome markers such as CD9, CD63 and CD81 are frequently used for EV detection, they may fail to capture the full spectrum of vesicle subtypes and may overlook placenta-specific subpopulations^42,43^. One of the most widely recognized and reliable placenta-specific surface markers is placental alkaline phosphatase (PLAP). This marker has been used in multiple studies to distinguish placental EVs from other circulating vesicles. In a longitudinal study by Sarker et al., the proportion of PLAP-positive (PLAP⁺) EVs in maternal blood was shown to increase progressively between gestational weeks 6 and 12, indicating a biologically regulated release and suggesting their potential utility as early biomarkers of pregnancy^38^. Similarly, Chen et al. used PLAP to isolate placental EVs from umbilical cord blood, reinforcing its practical value in EV identification during pathological pregnancies such as gestational diabetes mellitus (GDM)^41^. In another study, Miranda et al. used PLAP to examine associations between total and placental EV concentrations and clinical outcomes such as birth weight, further supporting its use in perinatal research^44^.

#### EV isolation and characterization methods

Multiple techniques are used to isolate and characterize EVs. The gold standard remains differential ultracentrifugation, involving sequential centrifugation steps (300–2,000*g* for cells/debris, 10,000*g* for apoptotic bodies and ~100,000*g* for EVs) followed by sucrose or iodixanol density gradient purification to improve purity^43^. Other techniques include: (1) size exclusion chromatography: separates based on hydrodynamic size^45^; (2) polymer-based precipitation: fast and user-friendly but may co-isolate contaminants^46^; (3) immunoaffinity capture: enables enrichment of specific EV subtypes using antibodies (for example, anti-CD63 and anti-PLAP)^47^; and (4) microfluidic platforms: allow label-based or label-free high-throughput EV isolation based on physical properties such as charge and size^48,49^. Given the variability of methods, the ISEV has issued standardized guidelines (MISEV2023) to enhance cross-study reproducibility^23^. In studies relevant to this Review, PLAP-based immunocapture has been widely used to selectively isolate placenta-derived EVs from maternal or cord blood, allowing focused characterization of their properties and potential biomarker functions^38,41,44^.

In summary, placental EVs are biologically active particles predominantly secreted by trophoblasts—particularly syncytiotrophoblasts—that serve as a critical interface between maternal and fetal tissues. These vesicles, typically ranging from 50 nm to 150 nm in size, are released into maternal circulation throughout pregnancy in a manner that is tightly regulated by gestational age and pathological conditions. Placental EVs can traverse the placental barrier bidirectionally, mediating molecular communication from fetus to mother and vice versa, thereby playing an active role in maternal–fetal signaling. The use of placenta-specific surface markers such as PLAP has enabled the selective identification and enrichment of placenta-derived EVs from biological fluids. Longitudinal studies have demonstrated dynamic changes in PLAP⁺ EV levels during early pregnancy, suggesting their potential relevance to pregnancy physiology. As functionally important messengers, placental EVs are emerging as key contributors to maternal and fetal adaptation during gestation. The next section will explore in greater detail how these vesicles influence maternal and fetal health outcomes.

### Placental EVs and maternal health and pregnancy outcomes

A successful pregnancy requires complex coordination between the maternal and fetal systems, involving immune tolerance^27^, vascular remodeling^28,29^ and metabolic adaptation^30,31^. Placental EVs are now recognized as essential mediators of this coordination, shaping maternal physiology through a wide array of signaling functions. These vesicles are dynamically released throughout gestation, with levels and cargo composition influenced by both developmental stage and pathological states^38,41,50^.

#### Gestational regulation of placental EV release

Placental EV production is tightly modulated by gestational age. Sarker et al. demonstrated that EV concentrations in maternal blood increase during the first trimester, suggesting a role in early placental signaling and maternal adaptation to pregnancy^38^. This developmental control is further indicated by the use of placenta-specific markers such as PLAP, which has been shown to increase between gestational weeks 6 and 12^38^. Kiu et al., using placental explants, estimated changes in EV secretion between weeks 8 and 12. While the number of EVs per milligram of explant tissue did not significantly change, accounting for the increase in placental weight revealed a substantial rise in the total amount of secreted EVs over the 4-week period^51^. These patterns support the view that placental EVs function as finely tuned messengers that reflect and help regulate the physiological state of pregnancy.

#### Maternal immune tolerance

One of the most remarkable adaptations in pregnancy is the maternal immune tolerance toward the semi-allogeneic fetus. This concept, first articulated by Peter Medawar, is supported by the phenomenon that fetal antigens can be present in maternal circulation without provoking immune rejection^52^. One mechanism contributing to this tolerance is the suppression of maternal immune cell proliferation and activity. Thibault et al. demonstrated that vesicles shed from the plasma membrane of syncytiotrophoblasts inhibited lymphocyte proliferation in vitro^53^. In addition, Mincheva-Nilsson et al. found that placental EVs carry major histocompatibility complex class I-related proteins A and B, which interact with receptors on natural killer cells to downregulate their activity—suggesting a mechanism for maternal immune evasion^54^. More broadly, placental EVs have been implicated in promoting this tolerance by delivering immunomodulatory molecules to maternal immune cells^27^. In vivo tracking studies using fluorescent reporter mice further support this function; placental EVs were shown to localize in maternal lungs and liver, where they interacted with both immune and non-immune cells^27^. Functional evidence was provided by the cleavage of fluorescent tags in recipient tissues, resulting in altered reporter signals and indicating direct modulation of cellular function^40^. Collectively, these findings suggest that placental EVs actively reprogram maternal immune responses, potentially inducing tolerogenic states that support fetal survival.

#### Pathological conditions during pregnancy

In pathological pregnancies, the quantity and composition of placental EVs are altered in ways that may contribute to disease pathogenesis. For instance, in GDM, Chen et al. found elevated levels of placental EVs in umbilical cord blood compared with normoglycemic pregnancies^41^. These EVs carry stress-responsive cargo, including microRNAs (miRNAs) and proteins reflective of the placenta’s metabolic state^31,55^, and may influence maternal insulin resistance or systemic inflammation. In preeclampsia, placental EVs exhibit heightened pro-inflammatory potential^56^. Ex vivo experiments using placental explants and maternal immune cells revealed that EVs from preeclamptic and early pregnancy samples induce stronger immune activation compared with those from term pregnancies^56^. Notably, preeclamptic EVs failed to induce lipopolysaccharide tolerance—a feature observed with EVs from normal pregnancies^56^—suggesting impaired immunomodulatory function and disrupted maternal immune adaptation. Infectious stressors can also modulate placental EV release. For example, Bergamelli et al. showed that HIPEC cytotrophoblast cells infected with human cytomegalovirus (HCMV) released significantly more EVs than uninfected controls^50^, suggesting that infectious triggers may amplify EV-mediated signaling and contribute to inflammatory complications.

**Fig. 1:**
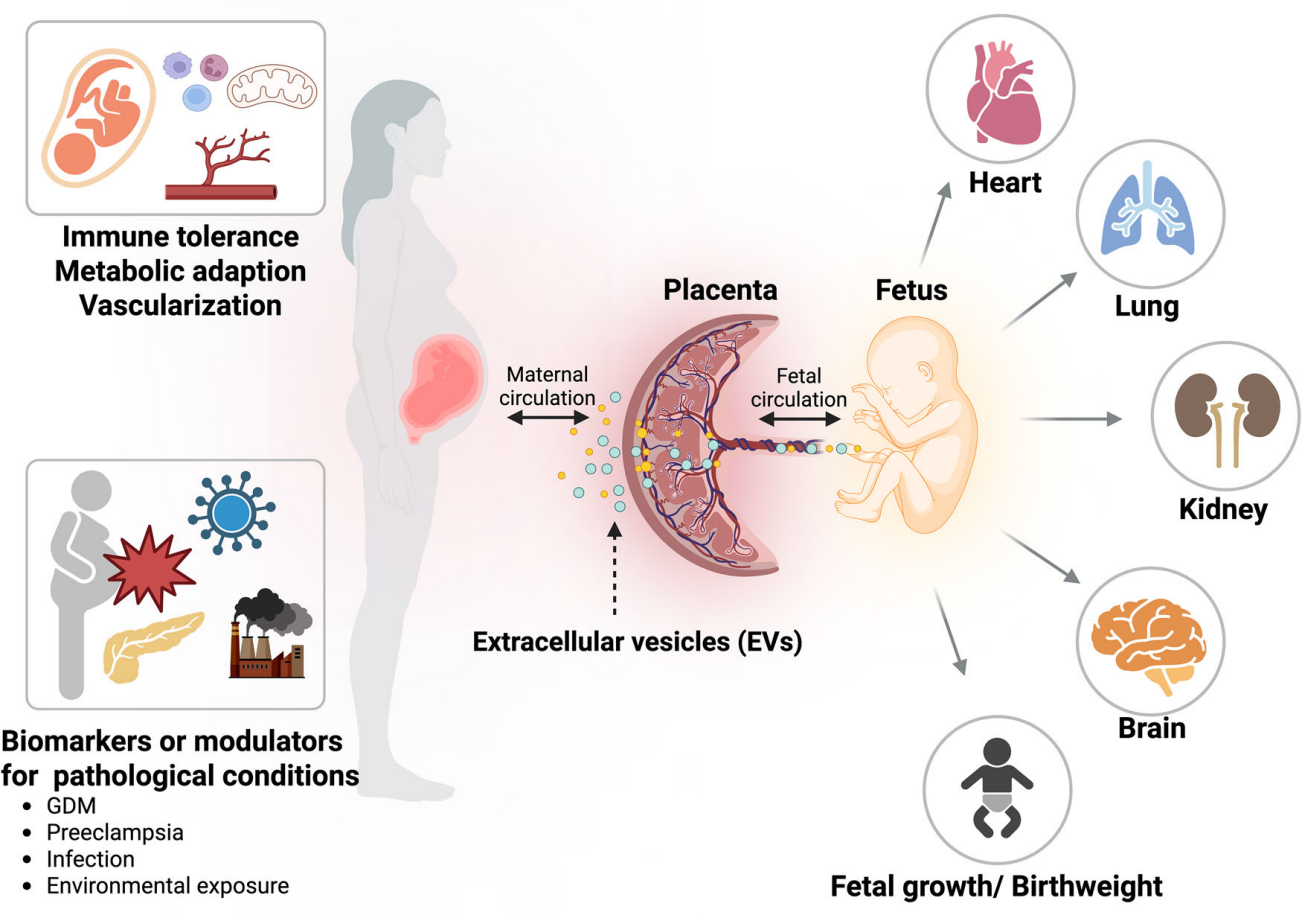
Placental EVs as mediators of maternal and fetal health. Placenta-derived EVs are actively secreted by trophoblasts and traverse the maternal–fetal interface to modulate both maternal and fetal physiology. In the maternal compartment, EVs contribute to immune tolerance, vascular remodeling and metabolic adaptation. In the fetal compartment, EVs influence organogenesis—including the heart, lungs and kidneys—and impact overall growth trajectories. These vesicles carry diverse cargo such as miRNAs, proteins and small molecules, and their composition dynamically responds to gestational stage and maternal environmental stressors, such as infection or metabolic dysregulation. The figure summarizes major sites of action and known roles of placental EVs in both maternal and fetal health outcomes. Figure created with BioRender.com.

In summary, placental EVs play a central role in orchestrating maternal adaptations necessary for a successful pregnancy (Fig. 1). They mediate immune tolerance, reflect the physiological state of the placenta and respond to pathological stimuli by altering their release and cargo composition. These vesicles are not only indicators but also potential contributors to conditions such as GDM and preeclampsia. Understanding their diverse functions provides critical insight into the mechanisms by which the placenta shapes maternal health. In the following section, we explore how these same vesicles influence fetal health and neurodevelopmental outcomes.

### Placental EVs and fetal health (non-neurological)

Placental EVs are increasingly recognized as active regulators of fetal organogenesis and growth, not merely as byproducts of placental turnover. By transferring bioactive cargo such as RNAs and proteins, these vesicles influence fetal development across multiple organ systems. The following sections highlight key studies supporting these roles (summarized in Table 2).

**Table 2:** Roles of placental EVs on fetal development and health outcomes.

Organ/outcomes	Model	Key findings	Ref
Heart	In vivo/ex vivo	Ovol2^+^ placental EVs promote cardiomyocyte maturation from placental tissue or differentiated trophoblast stem cells as well as increased heart rate and epicardial outgrowth.	^59^
Lung	In vitro/in vivo	GDM-EVs impair proliferation, branching and surfactant expression. GDM umbilical cord blood had greater numbers of EVs and had 61 dysregulated miRNAs.	^41^
Kidney	In vitro/in vivo	Oxidative stress EVs and preeclamptic EVs reduce vascular integrity, branching, tubular formation and barrier function.	^60^
Birth weight and growth	Human cohort	PLAP^+^ EVs positively correlated with fetal birth weight and lower PLAP^+^ ratio in fetal growth restriction.	^44^
Birth weight	Human cohort	From serum samples, miR-127-3p was inversely correlated with placental birth weight for gestational age in the second trimester and miR-26b-5p was positively associated with birth weight in the second trimester.	^61^
Congenital diaphragmatic hernia	Human cohort	Infants with congenital diaphragmatic hernia undergoing treatment who did not survive had elevated EV levels in amniotic and tracheal fluid. Four miRNAs were found to be significantly upregulated in EVs from nonsurvivors: miR-889-3p and miR-379-5p in amniotic fluid, and miR-223-3p and miR-503-5p in tracheal fluid.	^62^
Brain	In vitro	Human NPC exposure to iPS cell-derived trophoblasts shifted transcription from a neuronal to glial lineage specification and upregulated other pathways such as Wnt and NOTCH.	^66^
Brain	In vitro	Mouse iPS cell-derived trophoblasts showed subtype- and origin-specific changes on pathways such as neuronal polarity, axonal guidance and synaptic formation and also exhibited elevated levels of serotonin.	^67^

#### Cardiopulmonary development

The fetal heart and lungs are particularly susceptible to placental signals during the second trimester when rapid morphological differentiation occurs^57,58^. In a study by Jeyarajah et al., placental-specific deletion of Ovol2, a transcription factor expressed exclusively in placental tissue and not in fetal organs, resulted in defective fetal heart development and embryonic lethality^59^. The study elegantly demonstrated this by generating placenta-specific Ovol2-knockout mice using Tpbpa-Cre and observing severe cardiac hypoplasia and impaired cardiac contractility in embryos^59^. To dissect the mechanisms further, the researchers cultured fetal mouse hearts ex vivo in three conditions: (1) media conditioned by minced placental tissue, (2) media from Ovol2^+^ trophoblast stem cells and (3) media from Ovol2^−^ knockout trophoblasts. Only groups 1 and 2 significantly improved cardiomyocyte outgrowth and beating rate, indicating that Ovol2-dependent placental signals—probably mediated in part by EVs—are necessary for heart maturation. In the pulmonary system, Chen et al. investigated the effects of GDM-associated placental EVs using an integrative in vitro–in vivo approach^41^. EVs were isolated from maternal blood of women with GDM, and 61 miRNAs were found to be differentially expressed. When these EVs were applied to A549 human alveolar epithelial cells, there was a marked reduction in cell proliferation, upregulation of apoptotic markers, and suppression of surfactant proteins A–D, which are essential for lung function and gas exchange after birth. The study extended to ex vivo lung explant cultures and in vivo mouse models, where GDM-derived EVs led to disrupted alveolar branching morphogenesis, delayed lung maturation and abnormal lung morphology, all of which recapitulated features of neonatal respiratory distress often seen in GDM-affected pregnancies^41^.

#### Renal development

Gu et al. explored how placental EVs may affect fetal kidney morphogenesis, particularly under oxidative stress conditions that mimic preeclampsia^60^. EVs were isolated from normal and oxidative-stressed human trophoblasts and applied to primary human glomerular endothelial cells (HGECs)^60^. Compared with EVs from healthy controls, those from stressed trophoblasts significantly reduced HGEC proliferation, tube formation and barrier function, suggesting vascular toxicity^60^. When these EVs were applied to mouse fetal kidney explants, researchers observed impaired ureteric branching, decreased vascular endothelial cadherin and occludin expression, and aberrant nephron development^60^. Importantly, intra-amniotic injection of the same EVs into pregnant mice confirmed these defects in vivo^60^, supporting the hypothesis that placental EVs under stress conditions can directly disrupt nephrogenesis and renal vascularization.

#### Fetal growth and birth weight correlation

EVs have also been implicated in modulating global fetal growth trajectories. In a cohort study by Miranda et al., the ratio of placental EVs (PLAP^+^) to total EVs in maternal plasma was positively correlated with neonatal birth weight and placental weight^44^. The EV profiles were measured during mid-gestation, and the study concluded that a higher relative abundance of placental EVs may reflect placental robustness and enhanced nutrient delivery^44^. Similarly, an epidemiological study by Fudono et al. demonstrated trimester-specific associations between certain EV-derived miRNAs—miR-127-3p and miR-26b-5p—and fetal growth outcomes, including birth-weight-for-gestational-age and the placenta-to-fetal weight ratio^61^. These miRNAs included those involved in growth factor signaling and metabolic regulation^61^, highlighting a time-sensitive effect of EV cargo composition on fetal growth parameters. In more severe pathological contexts, elevated EV concentrations and altered miRNA profiles in amniotic fluid and fetal tracheal aspirates were associated with poor survival outcomes in fetuses with congenital diaphragmatic hernia undergoing fetal intervention^62^. Another study found that maternal plasma levels of placental EVs correlated positively with fetal growth parameters, supporting their potential as indicators of intrauterine growth and risk of growth restriction^44^. These findings further demonstrate that placental EVs can reflect fetal compromise and developmental risk.

In summary, placental EVs contribute directly to the structural and functional development of multiple fetal organ systems—including the heart, lungs, kidneys and overall somatic growth (Fig. 1). Their composition and bioactivity are sensitive to maternal metabolic and inflammatory states, such as GDM or oxidative stress. The tissue-specific targeting and dynamic regulation of their cargo underscore the functional role of placental EVs as active modulators of fetal organogenesis rather than passive correlates. These findings lay the groundwork for further investigation into how placental EVs may be leveraged to understand or potentially modulate fetal health trajectories.

### Placental EVs and fetal brain development

**Fig. 2:**
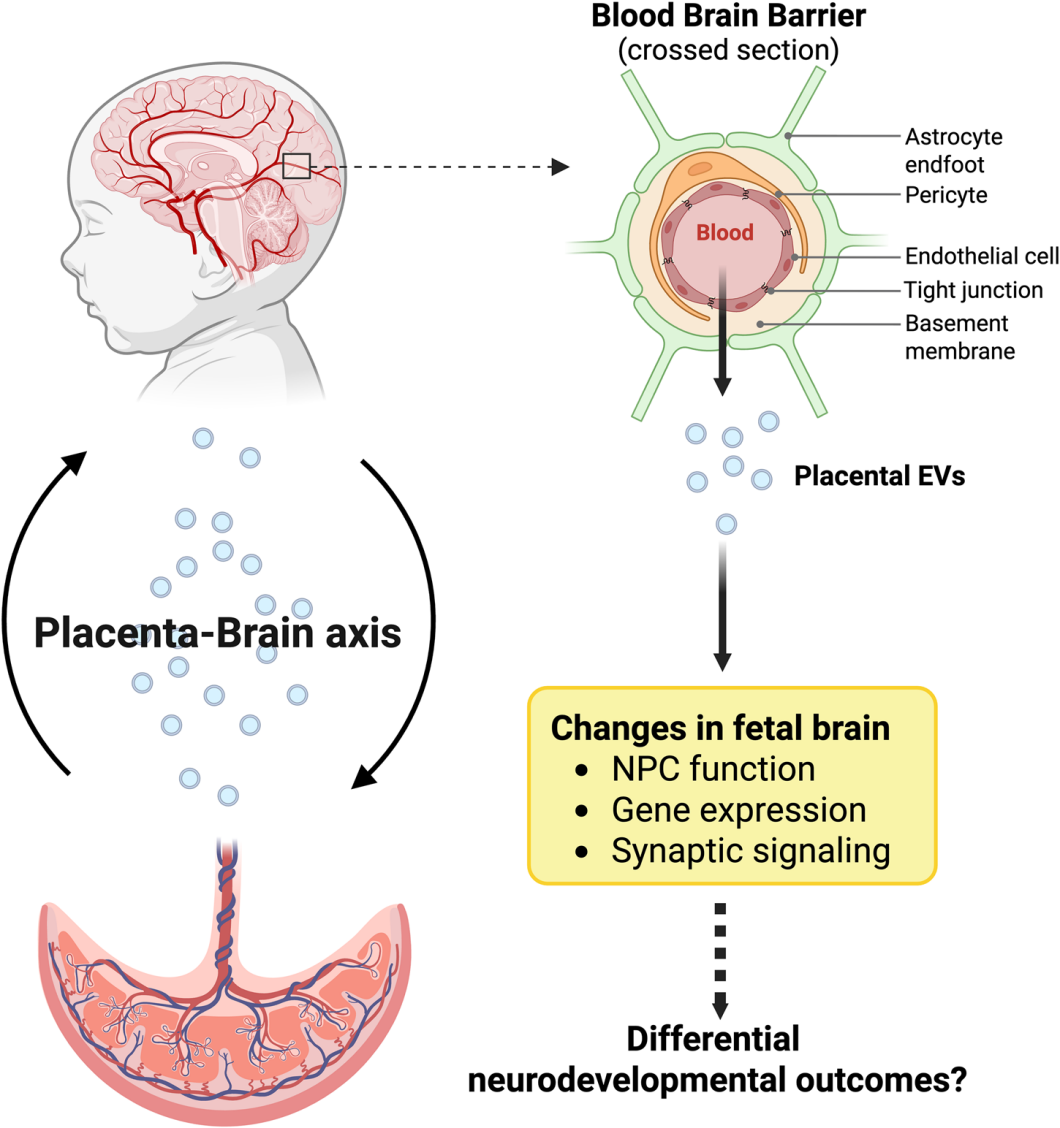
Role of placental EVs in fetal brain development and implications for neurodevelopmental outcomes. Emerging evidence supports a model in which placental EVs act as key regulators of fetal brain development. These vesicles are hypothesized to cross both the placental and fetal BBBs, delivering regulatory signals such as noncoding RNAs, proteins and neurotransmitters such as serotonin to NPCs. Experimental studies show that placental EVs modulate pathways involved in neurogenesis, regional brain patterning and cell fate specification—shifting neural progenitor transcriptomes toward glial or neuronal lineages and altering key developmental programs including Wnt, Notch and axon guidance signaling. The illustration depicts these putative routes and targets of EV-mediated communication in the developing fetal brain. Figure created with BioRender.com.

The fetal brain undergoes highly dynamic structural and functional changes during gestation, requiring precisely coordinated molecular signaling from both intrinsic and extrinsic sources. As vesicles capable of crossing both the placental barrier and the fetal blood–brain barrier (BBB), placental EVs are increasingly recognized as crucial intermediaries in placenta-to-brain communication, modulating early neurogenesis, regional patterning and cell fate decisions (Figs. 1 and 2).

Support for the role of EVs in fetal brain development first emerged from studies demonstrating their ability to reach the brain in vivo. In a pivotal study, EVs engineered with brain-targeting peptides and loaded with siRNA were injected into mouse circulation and later identified in brain tissue^63^. These EVs successfully mediated knockdown of specific mRNAs and proteins in the brain, providing compelling evidence that EVs can cross the BBB and influence neurological processes^63^. Further studies using placental EVs have shown their capacity to be internalized by endothelial barriers and trigger physiological changes. Notably, Cronqvist et al. demonstrated that placental EVs are predominantly taken up via clathrin-mediated endocytosis^64^ and that placental EVs traffic miRNAs to the mitochondria and endoplasmic reticulum of primary epithelial cells^65^. These findings provide proof of concept that EVs—potentially including placenta-derived EVs—can deliver regulatory cargo to neural tissues in vivo. The successful penetration of the BBB by EVs opens the possibility that endogenous placental EVs might exert similar physiological roles during normal brain development.

Mechanistic insights into how placental EVs affect fetal neural development have come from in vitro coculture models using human and mouse neural progenitor cells (NPCs). Kinkade et al. isolated EVs from human induced pluripotent stem (iPS) cell-derived trophoblasts, including both small and long noncoding RNA cargo, and applied them to human fetal NPCs^66^. Transcriptomic profiling of the recipient NPCs revealed that these EVs upregulated genes involved in forebrain patterning, including those regulating Wnt, Notch and glial fate pathways^66^. Interestingly, exposure to trophoblast EVs led to a transcriptional shift in NPCs from neuronal to glial lineage specification^66^, suggesting that EV cargo actively influences regional and cell-type specification in the fetal brain. A complementary study using mouse trophoblast-derived EVs showed that these vesicles altered NPC transcriptional programs in a subtype- and origin-specific manner^67^. For instance, EVs derived from trophoblast stem cells and differentiated syncytiotrophoblasts induced distinct transcriptomic effects on NPCs, particularly in pathways related to neuronal polarity, axonal guidance and synaptic formation^67^. These observations point to a nuanced role for placental EVs in region-specific and cell-type-specific neural differentiation.

Placental EVs appear to carry bioactive small molecules with direct neurodevelopmental relevance. One such molecule is serotonin, a neurotransmitter known to play a pivotal role in early brain development, particularly in forebrain formation. In a 2024 study, mouse trophoblast-derived EVs were found to contain elevated concentrations of serotonin compared to control EVs^67^. This aligns with earlier findings by Bonnin et al., which identified the placenta as a transient and critical source of serotonin during early gestation before the fetal serotonergic system becomes functional^68^. The ability of serotonin-loaded EVs to cross both the placenta and the BBB suggests a targeted delivery mechanism through which trophoblasts influence neurotransmitter signaling in the fetal brain. Such signaling may contribute to critical developmental processes including neuronal maturation, synaptogenesis and the early shaping of neural circuits that influence later cognitive and behavioral trajectories (Fig. 2).

Taken together, these findings support a model in which placental EVs act as active and multifaceted regulators of fetal brain development (summarized in Table 2). By trafficking across the placental and fetal BBB and delivering developmentally relevant molecular signals—including RNAs, proteins, and bioactive small molecules such as serotonin—placental EVs may influence key neurodevelopmental processes such as neuronal differentiation, regional patterning and synaptogenesis. However, despite growing mechanistic insights from in vitro and animal models, critical knowledge gaps remain. The precise mechanisms by which specific EV subtypes selectively target neural cells, the temporal windows of vulnerability or responsiveness, and the dose–response relationships of EV-mediated signaling are still poorly understood. Moreover, direct evidence of placental EV trafficking and functional impact on the human fetal brain is lacking, largely due to the inaccessibility of fetal neural tissues and ethical limitations. As a result, many of the proposed functions remain hypothetical in the human context, underscoring the need for innovative translational approaches and noninvasive biomarkers to bridge preclinical findings with clinical relevance. Future studies using human organoid models, advanced imaging and longitudinal cohort analyses will be essential to clarify the contribution of placental EVs to human brain development and disease susceptibility.

## Environmental exposures and placental EVs

The placenta serves as a dynamic interface between maternal and fetal environments and is highly responsive to external stressors^69^. A growing body of evidence suggests that placental EVs not only reflect these environmental insults but also participate in the signaling cascades that mediate their effects on fetal development. This section highlights how environmental exposures—including air pollution, infections and chemical toxicants—affect placental EV biology, with implications for fetal organogenesis and pregnancy outcomes (summarized in Table 3).

**Table 3:** Environmental exposures that alter placental EVs.

Exposure type	Model	EV alteration	Functional impact	Reference
Nanoparticles	Ex vivo	Anti-angiogenic shift	Impaired placental vascularization and functional changes linked to type of nanoparticle (metal oxides versus diesel exhaust particles)	^70^
PM_2.5_	Human cohort	miRNA profile changes	Linked to child neurodevelopmental delays in ASQ scores, communication and other behaviors at 6 months	^72^
Listeria infection	In vitro/in vivo	Pro-inflammatory EV RNA/proteins and RNA and nucleic acid binding proteins	Increased risk of reinfection (bacteria indirectly modulate this ability through EVs)	^76^
HCMV infection	In vitro/ex vivo	Viral protein-loaded EVs	Enhanced infection of fetal NPCs	^50^
BPA exposure	Rat model	EVs restored nutrient transporter expression (GLUT1)	Potential protective effect against toxicants	^77^
Cigarette smoke	In vitro	Induced cytokine-secreting EVs	Inflammation in fetal membranes (amnion epithelial cells and chorion trophoblast cells)	^78^

*ASQ* Ages & Stages Questionnaires.

### Air pollution and particulate matter

Airborne pollutants, particularly ultrafine particles and PM_2.5_, are among the most pervasive environmental threats during pregnancy and have been associated with adverse outcomes such as intrauterine growth restriction (IUGR), preterm birth and neurodevelopmental delays in children. Although the underlying mechanisms remain incompletely understood, placental EVs have emerged as likely intermediaries in this process. For instance, Dugershaw-Kurzer et al. exposed first-trimester placental explants to engineered nanoparticles, including metal oxides and diesel exhaust particles^70^. This exposure altered the placental secretome, with EVs showing anti-angiogenic profiles—such as reduced VEGF signaling and increased anti-angiogenic miRNAs^70^. These changes are particularly concerning given the critical role of early placental vascularization in supporting fetal oxygen and nutrient supply^71^. Perturbations in EV-mediated angiogenic signaling during this window could impair fetal organ development or contribute to IUGR^70^. A human cohort study by Wang et al. demonstrated that prenatal PM_2.5_ exposure was associated with neurodevelopmental delays in children, and these outcomes were correlated with differential miRNA expression in placental small EVs^72^. The altered miRNA profile included regulators of neurogenesis, synaptic signaling and oxidative stress responses^72^, underscoring the mechanistic role of EVs as transducers of pollutant-induced developmental toxicity.

### Pathogenic infections

Similar EV-based mechanisms are increasingly implicated in infection-related pregnancy complications. Maternal infections such as Zika virus^73^, cytomegalovirus^74^ and coronavirus disease 2019^75^ are well-known risk factors for adverse fetal outcomes, including microcephaly, sensorineural defects and increased perinatal mortality. Recent findings indicate that placental EVs play a role in pathogen-mediated signaling, potentially amplifying inflammatory responses or facilitating vertical transmission. In a model of *Listeria monocytogenes* infection, Kaletka et al. showed that infected mouse trophoblast stem cells secreted EVs with altered RNA and protein cargo, despite lacking direct bacterial protein contamination^76^. These EVs, when applied to macrophages, induced robust TNF-alpha secretion and heightened immune activation^76^. Interestingly, the same EVs increased susceptibility to repeat infection^76^, suggesting they may prime innate immune cells in a maladaptive manner. Meanwhile, HCMV was found to directly alter the protein composition of placental EVs^50^. Bergamelli et al. identified 31 viral proteins within HCMV-infected EVs, which were able to infect fetal NPCs in vitro and ex vivo^50^. This study provides direct evidence that EVs can act as carriers of viral components, facilitating placenta-to-brain transmission and increasing risk for fetal neuroinvasion and potential neurodevelopmental sequelae^50^.

### Chemical toxicants and endocrine disruptors

Chemical toxicants and endocrine disruptors, such as bisphenol A (BPA) and cigarette smoke, exert diverse and complex effects on placental physiology. In a rat model, Ermini et al. showed that BPA exposure decreased the expression of the glucose transporter (GLUT1) and the carnitine transporter (CPT1) in fetal myocardium^77^. Interestingly, when cardiac cells were treated with EVs isolated from BPA-exposed placental cells, these EVs reversed the suppression of GLUT1 and CPT1, restoring metabolic gene expression to near-normal levels. This suggests that EVs may have protective or compensatory roles, possibly acting as adaptive messengers that buffer against direct chemical toxicity. In another study, Shepherd et al. demonstrated that uterine epithelial cells exposed to cigarette smoke extract or TNF secreted EVs induced a pro-inflammatory cascade in amnion epithelial cells and chorionic trophoblasts^78^. These EVs elevated expression of cytokines such as IL-6 and TNF, suggesting that environmental stressors can modulate paracrine inflammatory signaling across fetal membranes via EVs^78^.

Together, these findings highlight that environmental toxicants can significantly alter placental EV biology—not only in terms of their cargo and secretion levels, but also in their downstream impact on fetal tissue signaling. Depending on the context, EVs may serve as adaptive mediators that mitigate toxicant effects or as pathogenic messengers that exacerbate inflammation and developmental risk.

## Future directions

Research on placental EVs has advanced substantially, progressing beyond descriptive studies into a dynamic field at the intersection of developmental biology, environmental health and translational medicine. As active mediators of maternal–fetal communication, placental EVs offer unique opportunities to uncover novel mechanisms of fetal development and to develop clinical tools for early diagnosis and intervention.

### Dissecting functional cargo and mechanisms

A key priority for future research is to establish the causal roles of specific EV cargo—such as miRNAs, long noncoding RNAs, proteins and neuroactive small molecules such as serotonin—in directing fetal outcomes. While existing studies have documented correlations between EV cargo profiles and developmental phenotypes, direct functional validation remains limited. Future work should aim to uncover how individual cargo components modulate recipient cell signaling in a context- and tissue-specific manner. This could be achieved through integrative functional approaches that combine molecular perturbation with high-resolution mapping of downstream cellular responses across developing fetal tissues.

### Advancing EV classification and standardization

Another critical challenge lies in the refinement of EV classification systems. Current nomenclature—based on vesicle size and presumed biogenesis—fails to capture the heterogeneity of placental EV populations. This is particularly problematic in the placental context, where vesicles may originate from different trophoblast subtypes and vary dramatically in content and function. Building upon emerging guidelines such as MISEV2023^23^, future classification efforts should incorporate molecular cargo, surface markers and cell of origin to better distinguish functionally distinct EV subsets, including those derived from syncytiotrophoblasts, cytotrophoblasts and fetal tissues.

### Technological innovation in tracking and functional studies

Emerging tools now enable real-time visualization and functional dissection of EV activity in vivo. These include fluorescent EV reporter mice (for example, CRE-loxP systems for cargo tracking)^40^, bioluminescent vesicle labeling for noninvasive imaging, and microfluidic EV chips for high-throughput cargo profiling^49^. Engineered vesicles—such as ARMMs—further expand the potential of EVs as delivery platforms for therapeutic molecules^16,17,79,80^. Numerous studies have utilized engineered vesicles through protein loading, hypoxic cell preconditioning or genetic modulation to promote tissue repair or treat diseases, including regeneration of tendons^81^ and skin^82^, among others^83^. Preclinical work has also demonstrated the ability of EVs to deliver antiviral proteins, such as IFITM3, across the placenta to protect the fetus^84^. However, direct evidence of placental EV transfer to the fetus in humans remains sparse. Most mechanistic insights come from rodent models or in vitro systems. To bridge this gap, the development of advanced human-relevant models—such as maternal–fetal interface organoids, placenta-on-a-chip devices and in vivo imaging techniques—will be essential to determine whether EV trafficking and function observed in animal models are recapitulated in human pregnancy.

### EVs as biomarkers and interventional tools

Placental EVs also hold promise as early, noninvasive biomarkers of pregnancy health. They are detectable in maternal blood as early as the first trimester and reflect gestational age, placental condition and exposure to stressors. For example, PLAP^+^ EVs and miRNA cargo could be used to predict birth weight or risk of IUGR^44^. In addition, EV-based ‘liquid biopsies’ may detect fetal neurodevelopmental risk long before clinical symptoms appear^85^. Integrating EV profiling with multi-omics technologies—including proteomics, metabolomics and epigenomics—could yield sensitive biomarker signatures for fetal growth restriction, preterm birth risk or neurodevelopmental impairment. These advances may allow clinicians to detect fetal compromise or developmental delay long before clinical symptoms arise.

### Integrating environmental exposure into EV research

Understanding how environmental exposures reshape EV biology is of growing importance. Exposure to air pollution, infections and chemical toxicants alters EV cargo, release dynamics and downstream signaling, with implications for fetal programming and long-term health^70,72–78^. Future studies should define the temporal and dose-dependent characteristics of these EV responses, assess whether the changes are adaptive or maladaptive, and investigate how they interact with maternal physiology to affect fetal outcomes. Importantly, EVs may act not only as messengers of environmental harm but also as compensatory regulators, and distinguishing these dual roles will be essential for identifying meaningful targets for intervention.

Together, these research directions will clarify the functional roles of placental EVs in fetal development, improve the reliability of EV-based diagnostics and unlock their potential as therapeutic tools. Bridging the current gaps between animal models, in vitro systems and human biology will be critical to ensure that placental EV research translates into meaningful improvements in maternal and child health.

## Conclusion

Over the past decade, placenta-derived EVs have emerged as dynamic and multifunctional mediators of intrauterine signaling. Once regarded as cellular waste, these nanoscale vesicles are now recognized as central to maternal–fetal communication, capable of modulating immune function, vascular remodeling, organogenesis and even neurodevelopment. They carry diverse molecular cargo—including RNAs, proteins and bioactive metabolites—that reflect the physiological or pathological state of the placenta and have the potential to influence fetal health at a distance.

Placental EVs can traverse both the maternal–fetal interface and the BBB, potentially enabling them to deliver regulatory molecules such as serotonin to the developing brain. Under maternal stress conditions, including gestational diabetes or environmental exposures, these vesicles exhibit altered cargo and signaling patterns, suggesting a role in transmitting environmental influences to the fetus. Despite growing evidence from animal and in vitro studies, important gaps remain—particularly regarding their functional relevance in humans. The mechanisms of EV release, tissue targeting and direct transfer to fetal organs remain to be fully elucidated, underscoring the need for advanced human-relevant models and imaging technologies.

Looking ahead, placental EVs are poised to become powerful tools in both basic and clinical science. Their accessibility in maternal biofluids, molecular richness and responsiveness to physiological changes make them ideal candidates for noninvasive biomarkers of fetal well-being. Moreover, their potential as therapeutic vectors opens new possibilities for targeted fetal intervention. As research advances, interdisciplinary collaboration will be key to translating these insights into clinical practice. In an era increasingly defined by personalized and preventative medicine, placental EVs offer a promising avenue to better understand and safeguard fetal development—laying the foundation for healthier beginnings.
